# Supplementary material for: Grain filling in barley relies on developmentally controlled programmed cell death
Source: Commun Biol. 2021 Mar 30;4:428. doi: 10.1038/s42003-021-01953-1 (PMC8009944; doi:10.1038/s42003-021-01953-1)
Supplement: Supplementary file 2 — Supplementary Information [file 42003_2021_1953_MOESM2_ESM.pdf]

Supplementary Information

Grain filling in barley relies on developmentally controlled programmed cell death

Volodymyr Radchuk*, Van Tran, Alexander Hilo, Aleksandra Muszynska, Andre Gündel, Steffen Wagner, Joerg Fuchs, Goetz Hensel, Stefan Ortleb, Eberhard Munz, Hardy Rolletschek, Ljudmilla Borisjuk*

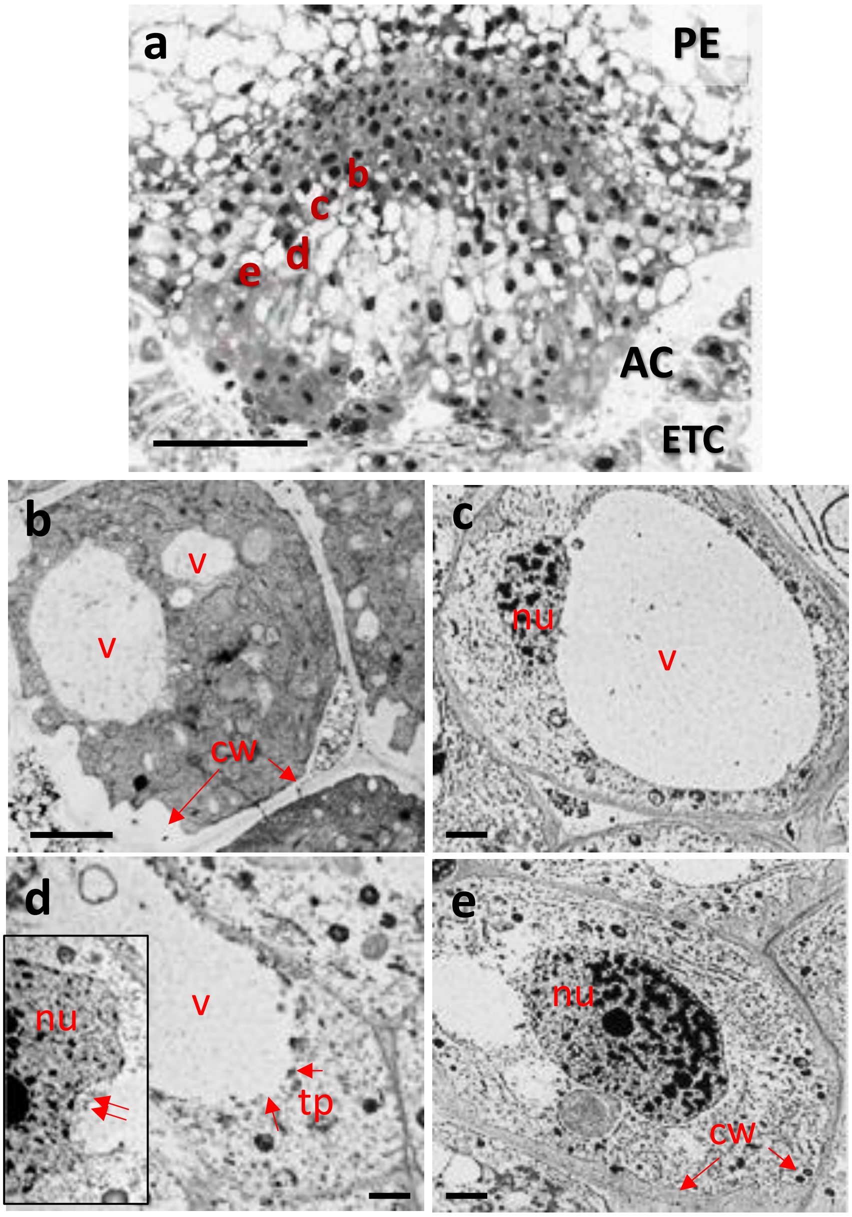

Supplementary Fig. 1. Gradient of PCD in the NP of the developing barley grains. a, A general view of the nucellar projection (8 DAF). Individual cells along of disintegration gradient are marked by red letters and shown in **b-e**. **b**, A differentiated cell, forming large vacuole, with thick invaginated cell walls and small vacuoles. **c**, An elongating cell with a large vacuole and condensing nucleus. **d**, Rupture of the tonoplast membrane (red arrowed). Advanced vacuolar disintegration and the rupture of the nuclear membrane (double red arrowed) is visible in the neighbour cell (left). **e**, A cell with the condensed nucleus, only small vacuolar rests are visible. Bars = 100 µm in **a**, and 15 µm in **b-e**. Abbreviations: ac, apoplastic cavity; cw, cell wall; ETC, endosperm transfer cells; NP, nucellar projection; nu, nucleus; PE, pericarp tp, tonoplast; v, vacuole.

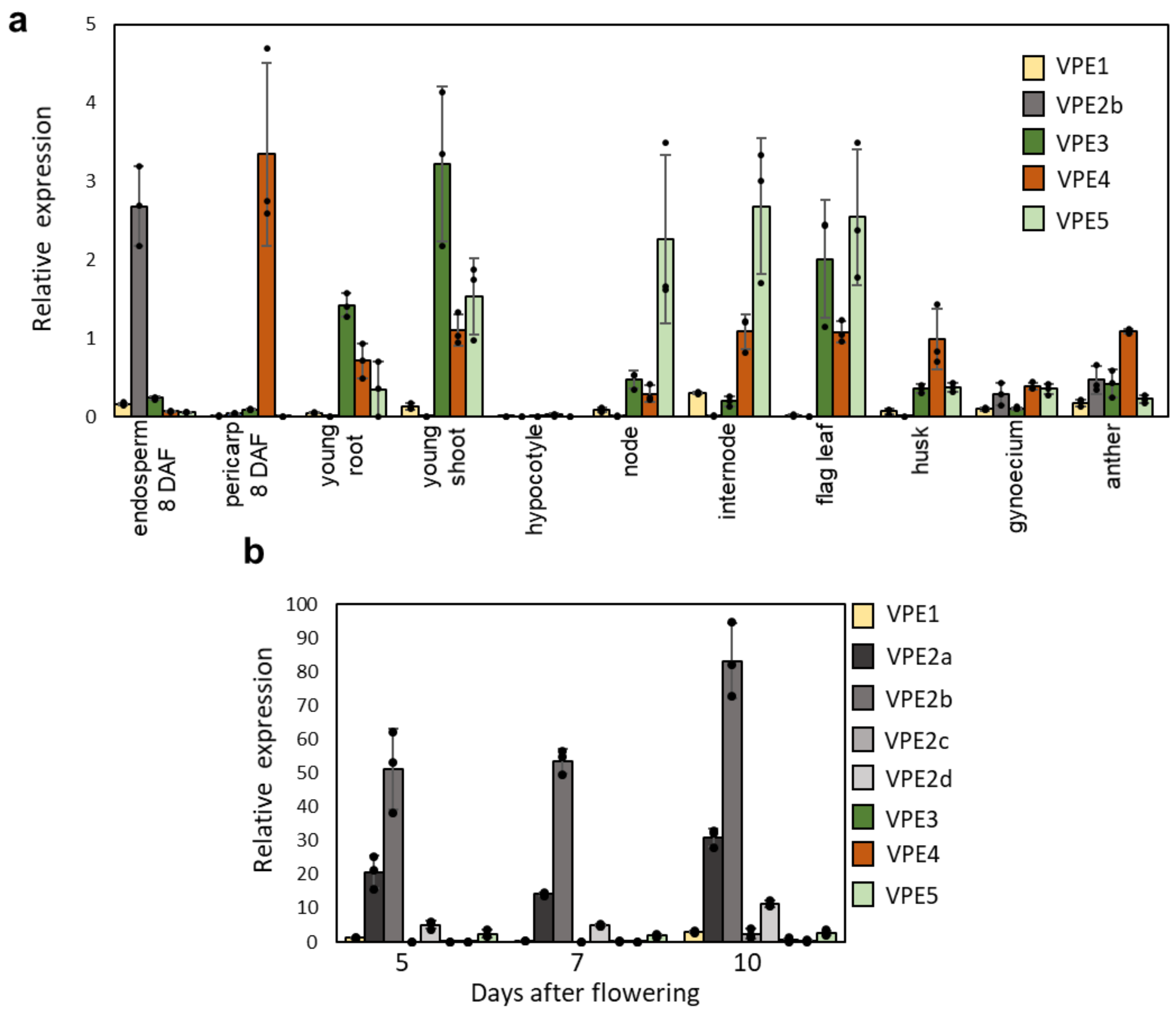

Supplementary Fig. 2. The expression of the VPE gene family in the different barley tissues (**a**) and in the micro-dissected nucellar projection of the developing barley grain (**b**) assessed using qRT-PCR. Nucellar projection was micro-dissected as described in Tran et al., 2014. Data are shown as the mean ± SD (n = 3).

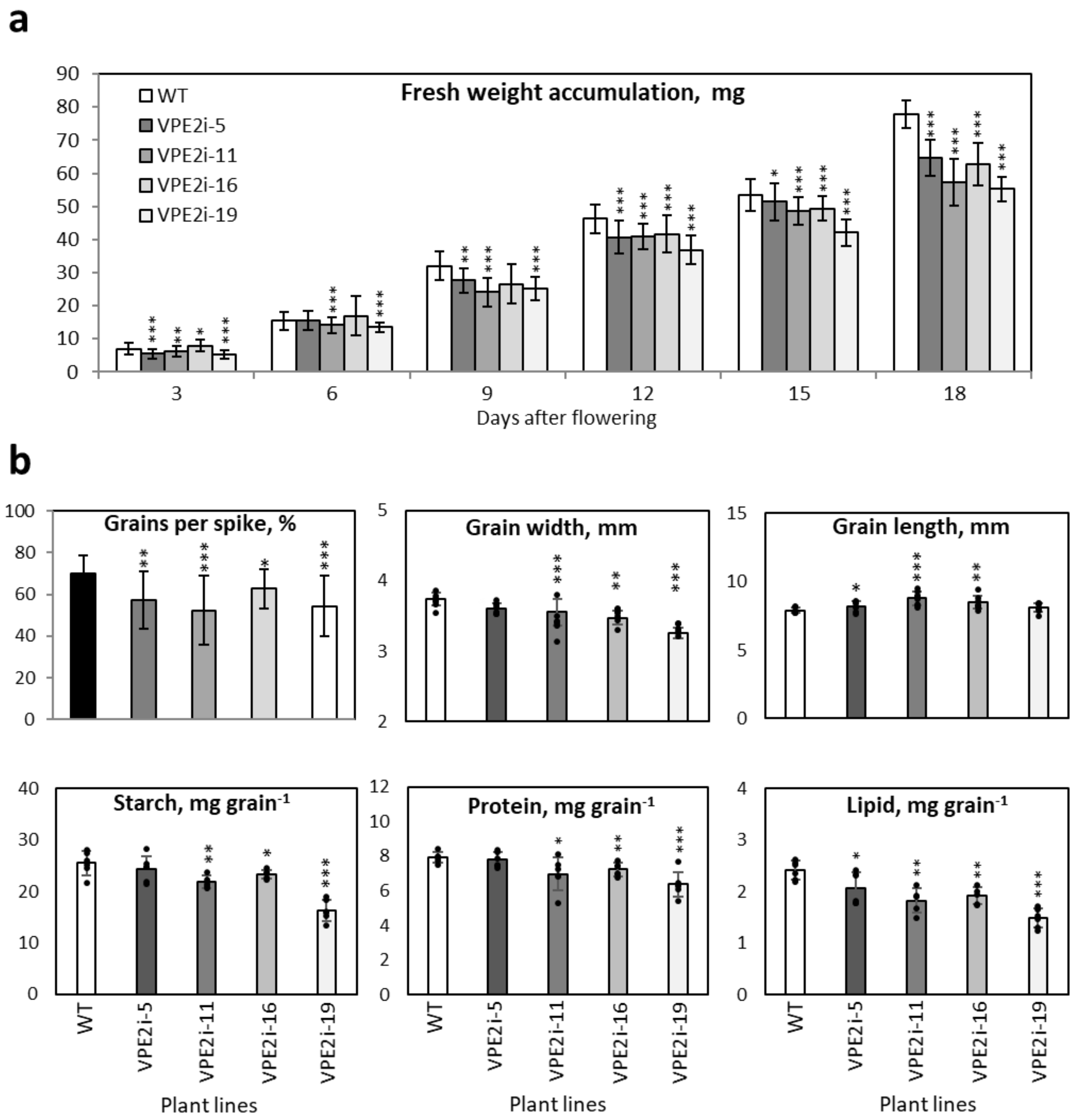

Supplementary Fig. 3. Phenotypic characters of the VPE2 subfamily-repressed grains. a, Accumulation of fresh weight by the developing transgenic and WT grains; **b**, Characteristics measured at maturity: grains per spike, grain width, grain length, content of starch, protein and lipid in the mature transgenic grains compared to the WT. Data are presented as the mean ± SD, n = 40-80 for **a, =** 20 for grains per spike, = 8 for grain width and length, and = 6 for starch, protein and lipid contents in **b**; *, **, ***: means differ at $P < 0.05$, < 0.01 and < 0.001, respectively.

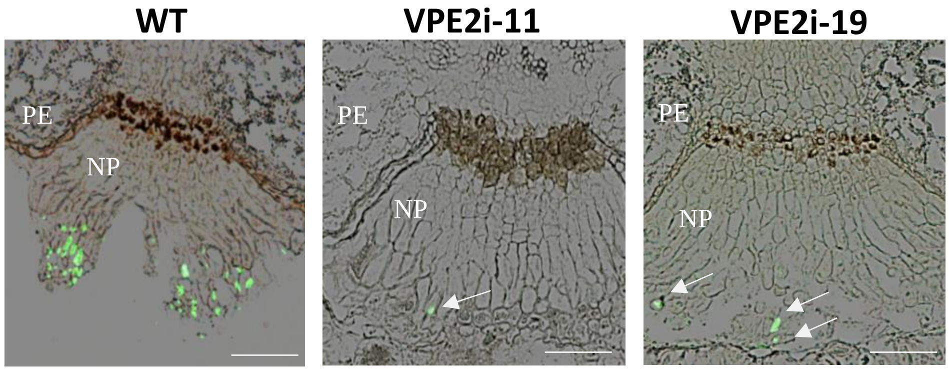

Supplementary Fig. 4. A TUNEL assay visualizing nuclei degradation in nucellar projection of WT (left), VPE2i-11 (middle) and VPE2i-19 (right) grain. Numerous degrading nuclei at margins of NP are visible in WT (green labelling) while nucleus degradation is almost absent in the transgenic lines (arrowed). Bar: 50 µm. Abbreviations: NP, nucellar projection; PE, pericarp, WT, wild type.

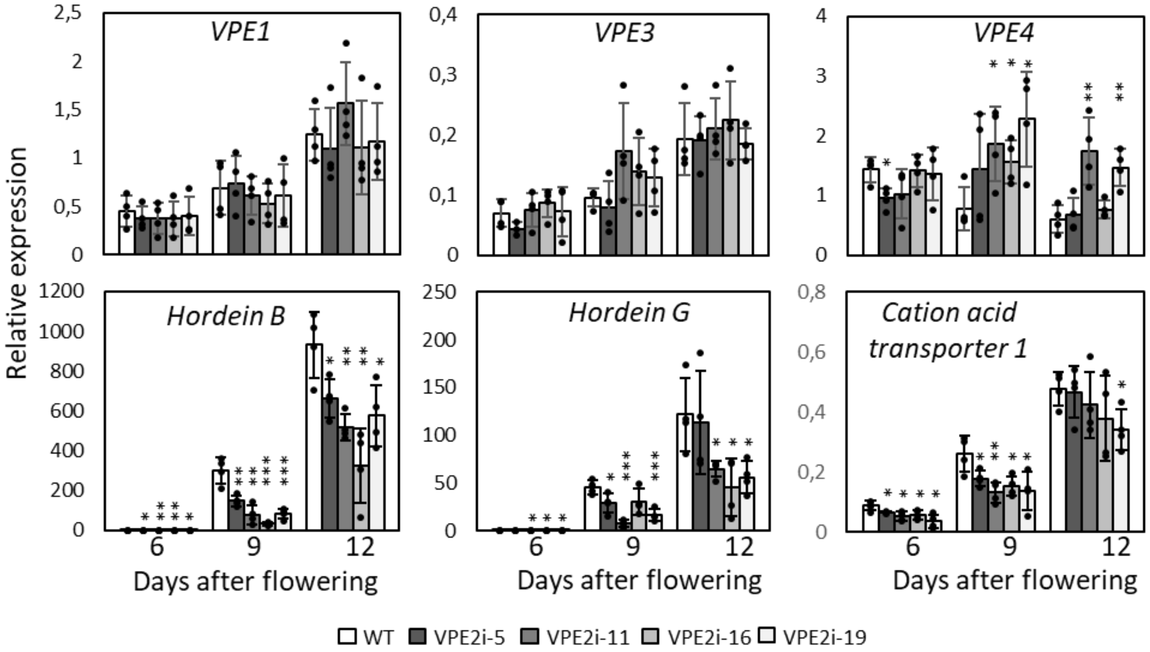

Supplementary Fig. 5. Transcript abundancies of some selected genes in *VPE2a-d* repressed grains based on qRT-PCR. Data are shown as the mean ± SD (n = 4). *, **, ***: means differ at $P < 0.05$, < 0.01 and < 0.001, respectively.

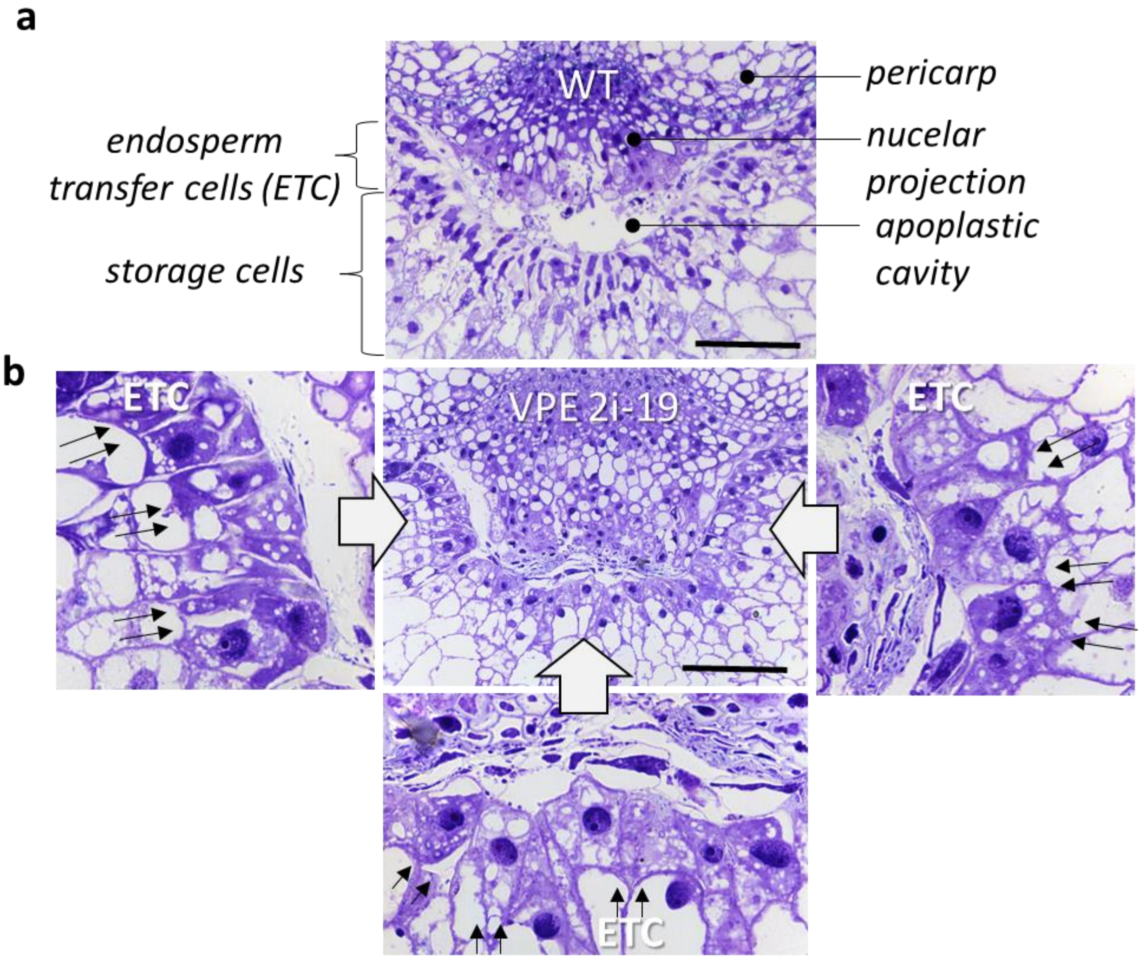

Supplementary Fig. 6. The morphology of the ETCs, formed in the grain of WT (**a**) and transgenic line VPE2i-19 (**b**) at 8 days after flowering. **a**, Typical densely packed ETC layer in WT; **b,** The corresponding region in the VPE2i-19 transgenic line; large arrows point to the enlarged region, showing details; double arrows point to the ETC. Bars = 100 μm.

Supplementary Table 2. List of primers used for qRT-PCRs.

Gene name	Gene ID	forward primer	reverse primer	fragment length
amino acid permease 3 (AAP3)	HORVU7Hr1G039670	5'-AACCCTTTTCCTATTCGCCGCC-3'	5'-CAAAACCACACCTGCCTGCTAC-3'	150
actin	HORVU1Hr1G002840	5'-CTGGTTTCGCTGGAGATGATGC-3'	5'-CCGAGGGCGACCAACTATGC-3'	62
ADP-Glucose transporter	AAT12275.2	5'-TACGCGGCGGCCAACTTCTAC-3'	5'-GCTTCCGCGCCACCTCCAG-3'	160
Small subunit 1a of AGPase (AGP-S1a)	HORVU7Hr1G067620	5'-GCCGCCTCCCCTTCCAAGAT-3'	5'-CGGCACGTGGGGAGAAGAAGA-3'	181
BETL	HORVU7Hr1G106040	5'-CCCAAGTTTGCCACGGTTACA-3'	5'-TAGGCGCCTTCGAGTGGTGGAG-3'	116
cationic amino acid transporter (CAT1)	HORVU5Hr1G006900	5'-ATGGATTCTGGCACGCCATGAG-3'	5'-AGGAAAACCAAGGCAGCAGCAC-3'	75
cathepsin B1	HORVU4Hr1G010360	5'-TACCTCCTCCGCTTGCTTCCTC-3'	5'-GGCCGCTCCCCATCTTCGTC-3'	71
END1	HORVU0Hr1G026630	5'-GGCGGATGCGACGGTGAT-3'	5'-ATGGGATGTTCGCCTTCTGC-3'	127
Hordein B	HORVU1Hr1G001140	5'-CCACAACAAACACCACTCCCAC-3'	5'-TGCAACACATGGCAACTGCTC-3'	197
Hordein D	HORVU1Hr1G064080	5'-CAAGGACAAGGGCAACAAGGAC-3'	5'-ACTGCTGCGGAGAAATTGCAC-3'	103
Hordein G	HORVU1Hr1G000700	5'-CCCACACAACAATTCCCCCAAC-3'	5'-TGTAACGGTGGCTGAGGGTATG-3'	137
Granule bound starch synthase 1a (GBSS1a)	HORVU7Hr1G012380	5'-TGCGGCGCTTGGTATGAGGACT-3'	5'-GGCGCCGACGAACACGAG-3'	139
Granule bound starch synthase 1b (GBSS1b)	HORVU2Hr1G090980	5'-GGGTTCCACATGGGTTCGTTCA-3'	5'-CACTTCTTTGCCGGTCCCTTCC-3'	176
Jekyll1	AM261729	5'-TTTGGGTGCCGGAGGAAGAGTA-3'	5'-CACCCCCGGCAGGAACAGC-3'	156
MADS29	HORVU6Hr1G032220	5'-GGGCGGGCTGCTCAAGAAGG-3'	5'-CTGGAGGCGGGGCTGGAGTA-3'	114
Sucrose transporter 1 (SUT1)	HORVU4Hr1G075200	5'-GGCGGCCATCTGCATCATAAGC-3'	5'-GGCGAAGAGGGCGAGGGAGAC-3'	112
Sucrose transporter 2 (SUT2)	HORVU5Hr1G000010	5'-GGGAACGCTGCCTACACCACAC-3'	5'-GGGCCCACGGAGGAAAGTAACA-3'	145
SWEET11a	HORVU5Hr1G076770	5'-GTCGCCGCCTTCTCCCTCAT-3'	5'-GTACTCGGCGCTCTTGGTCTTG-3'	162
SWEET11b	HORVU7Hr1G054710	5'-AACGTGGGCGGCTTCTTCTTCA-3'	5'-CGGCGTCGGCGGTAGTAGTG-3'	100
vacuolar processing enzyme 2a (VPE2a)	HORVU2Hr1G091880	5'-TGCGCTGCAGTACACGGAA-3'	5'-TCTAGCTAGCTAGGAACCTCCG-3'	57
vacuolar processing enzyme 2b (VPE2b)	HORVU2Hr1G092090	5'-CAGCGCTTGCAACGGCTACGA-3'	5'-TGCAAGCGGATCAGGGCTGTG-3'	72
vacuolar processing enzyme 2c (VPE2c)	HORVU2Hr1G092080.6	5'-GCGTCTCTGAGGCCCAAATGA-3'	5'-TTATAACCGCCGCAAGCACTGAT-3'	51
vacuolar processing enzyme 2d (VPE2d)	HORVU2Hr1G092080.15	5'-GCTGCCTTTGCCCATCCTG-3'	5'-TCCCCCGTTTAACTGCTCATACTT-3'	91

Supplementary Table 3. Chromatographic and mass spectrometry conditions applied to analyze metabolite pattern in immature barley grains.

**IC-MS-based untargeted profiling of anionic central metabolites**		**UHPLC-MS-based untargeted profiling of cationic metabolites**	
**Analytical columns:**<br>Dionex™ IonPac™ AS11-HC-4µm (2x250 & 2x50mm)<br>**Eluents:**<br>A: H_2O<br>B: 100mM KOH (generated by Dionex EGC 500 KOH cartridge)<br>**Gradient:**<br>**Time** **%B**<br>0-12 10-25<br>12-15 25-100<br>15-28 100<br>28-28.5 100-10<br>28.5-32 10<br>**Flow parameters:**<br>Flow 0.35 mL/min<br>Temp 35 °C<br>Inj. Vol 10 µL	**MS-Tune parameters:**<br>Polarity: negative<br>Spray voltage: 3.5 kV<br>Sheath gas flow: 36<br>Aux. gas flow: 5<br>Capillary temp.: 320 °C<br>Aux. gas temp.: 325 °C<br>S-lens: 50<br>**Mode: Full MS**<br>Time: 0-30 min<br>Resolution: 140 K<br>m/z Range: 67 - 1000<br>Inject time: 200 ms<br>Auto gain control: 3e6<br>**Mode: Full MS / ddMS2**<br>Time: 0-30 min<br>FullMS:<br>Resolution: 70 K<br>m/z Range: 67 - 1000<br>Inject time: 100 ms<br>Auto gain control: 1e6<br>dd-MS2:<br>Resolving power: 17.5 K<br>Inject time: 50 ms<br>Auto gain control: 1e5<br>Loop count: 5<br>Collision energy: 15; 25; 35	**Analytical columns:**<br>Column: Hypersil Gold, 1.9µm (2.1x150mm)<br>**Eluents:**<br>A: H_2O + 0.1% Formic acid (FA)<br>B: MeOH + 0.1% FA<br>**Gradient:**<br>**Time** **%B**<br>0-2 0<br>5-9 15-98<br>9-14 15-98<br>14-15 98-0<br>15-18 0<br>**Flow parameters:**<br>Flow 0.4 mL/min<br>Temp 40 °C<br>Inj. Vol 1 µL	**MS-Tune parameters:**<br>Polarity: positive<br>Spray voltage: 4 kV<br>Sheath gas flow: 30<br>Aux. gas flow: 5<br>Capillary temp.: 320 °C<br>Aux. gas temp.: 325 °C<br>S-lens: 50<br>**Mode: Full MS**<br>Time: 0-16min<br>Resolving power: 140 K<br>m/z Range: 63 - 945<br>Inject time: 200 ms<br>Auto gain control: 3e6<br>**Mode: Full MS / ddMS2**<br>Time: 0-16 min<br>FullMS:<br>Resolution: 35 K<br>m/z Range: 63 - 945<br>Inject time: 50 ms<br>Auto gain control: 1e6<br>dd-MS2:<br>Resolution: 17.5 K<br>Inject time: 50 ms<br>Auto gain control: 1e5<br>Loop count: 5<br>Collision energy: 30

Supplementary Table 4. Accession numbers of VPEs from *Brachypodium*, barley, rice, wheat and maize used in the study.

species	Gene name	Gene ID	Other gene names
Aegilops speltoides	*AesVPE1*	XP_020155210	
	AesVPE2a	XP_020167168	
	AesVPE2b	XP_020198904	
	AesVPE2c	XP_020189920	
	AesVPE2d	XP_020167160	
	AesVPE3	XP_020175758	
	AesVPE4	XP_020188162	
	AesVPE5	XP_020180174	
	AesVPE6	XP_020180175	
Brachypodium distachyon	*BdVPE1*	BRADI_3g50100v3	
	BdVPE2	BRADI_5g16960v3	
	BdVPE3	BRADI_2g41270v3	
	BdVPE4	BRADI_4g30110v3	
Hordeum vulgare	*HvVPE1*	FR696360	HvLeg1
(barley)	*HvVPE2a*	FR696361	Nucellain, HvLeg6
	HvVPE2b	FR696362	HvLeg2
	HvVPE2c	FR696363	HvLeg7
	HvVPE2d	FR696364	HvLeg3
	HvVPE3	FR696365	HvLeg4
	HvVPE4	FR696366	HvLeg5
	HvVPE5	BAJ95226	HvLeg8
Oryza sativa	*OsVPE1*	Os04t0537900	GLUP3
(rice)	*OsVPE2*	Os01t0559600	
	OsVPE3	Os02t0644000	
	OsVPE4	Os05t0593900	
	OsVPE5	Os06t0105100	
Triticum aestivum	*TaVPE1A*	TraesCS6A02G238500	
(common wheat)	*TaVPE1B*	TraesCS6B02G285900	
	TaVPE1D	TraesCS6D02G220900	
	TaVPE2aA	TraesCS2A02G386600	
	TaVPE2aB	TraesCS2B02G404200.1	
	TaVPE2aD	TraesCS2D02G383400.1	
	TaVPE2bA	TraesCS2A02G093500LC.1	
	TaVPE2bB	TraesCS3B02G346000.1	
	TaVPE2bD	TraesCS7D02G469900LC.1	
	TaVPE2cA	TraesCS2A02G386700.1	
	TaVPE2cB	TraesCS2B02G404300.1	
	TaVPE2cD	TraesCS2D02G383500.1	
	TaVPE2dA	TraesCS2A02G385900.1	
	TaVPE2dB	TraesCS2B02G402800.1	
	RaVPE2dD	TraesCS2D02G382400.1	
	TaVPE3A	TraesCS3A02G204500.1	
	TaVPE3B	TraesCS3B02G233000.2	
	TaVPE3D	TraesCS3D02G534100.2	
	TaVPE4A	TraesCS5A02G228900.1	
	TaVPE4B	TraesCS5B02G227600.1	
	TaVPE4D	TraesCS5D02G238800.1	
	TaVPE5A	TraesCS3A02G528900.1	
	TaVPE5B	TraesCS3B02G597800.1	
Zea mays	*TaVPE5D*	TraesCS3D02G534200.2	
(maize)	*ZmVPE1*	Zm00001d017399	
	ZmVPE2	Zm00001d002824	
	ZmVPE3	Zm00001d011174	
	ZmVPE4	Zm00001d009759	
	ZmVPE5	Zm00001d009759	
