# Supplementary material for: Grain filling in barley relies on developmentally controlled programmed cell death
Source: Commun Biol. 2021 Mar 30;4:428. doi: 10.1038/s42003-021-01953-1 (PMC8009944; doi:10.1038/s42003-021-01953-1)
Supplement: Supplementary file 3 — Description of Additional Supplementary Files [file 42003_2021_1953_MOESM3_ESM.pdf]

Description of Additional Supplementary Files

File name: Supplementary Data 1

Description: Untargeted metabolite analysis of developing grains in response to suppression of *VPE2a*-*VPE2d* genes.

File name: Supplementary Data 2

Description: Source Data.

File name: Supplementary Movie 1

Description: MRI-based three-dimensional view of the structure of a developing barley grain attached to a spike. Reference (light microscopic) image explains the positioning of endosperm, pericarp, NP and apoplastic cavity.

File name: Supplementary Movie 2

Description: Three-dimensional comparative view of the grains from WT and the VPE2i-11 transgenic line as visualized by MRI.

File name: Supplementary Movie 3

Description: Three-dimensional comparative view of the grains from WT and the VPE2i-19 transgenic line as visualized by MRI.
