# Supplementary material for: Grain filling in barley relies on developmentally controlled programmed cell death
Source: Commun Biol. 2021 Mar 30;4:428. doi: 10.1038/s42003-021-01953-1 (PMC8009944; doi:10.1038/s42003-021-01953-1)
Supplement: Supplementary file 9 — Reporting Summary [file 42003_2021_1953_MOESM9_ESM.pdf]

nature research

Corresponding author(s): Volodymyr Radchuk, Ljudmilla Borisjuk

Last updated by author(s): Feb 17, 2021

Reporting Summary

Nature Research wishes to improve the reproducibility of the work that we publish. This form provides structure for consistency and transparency in reporting. For further information on Nature Research policies, see our Editorial Policies and the Editorial Policy Checklist.

Statistics

For all statistical analyses, confirm that the following items are present in the figure legend, table legend, main text, or Methods section.

n/a	Confirmed	
☐	☒	The exact sample size (n) for each experimental group/condition, given as a discrete number and unit of measurement
☐	☒	A statement on whether measurements were taken from distinct samples or whether the same sample was measured repeatedly
☒	☐	The statistical test(s) used AND whether they are one- or two-sided<br>*Only common tests should be described solely by name; describe more complex techniques in the Methods section.*
☒	☐	A description of all covariates tested
☐	☒	A description of any assumptions or corrections, such as tests of normality and adjustment for multiple comparisons
☐	☒	A full description of the statistical parameters including central tendency (e.g. means) or other basic estimates (e.g. regression coefficient) AND variation (e.g. standard deviation) or associated estimates of uncertainty (e.g. confidence intervals)
☐	☒	For null hypothesis testing, the test statistic (e.g. F, t, r) with confidence intervals, effect sizes, degrees of freedom and P value noted<br>*Give P values as exact values whenever suitable.*
☒	☐	For Bayesian analysis, information on the choice of priors and Markov chain Monte Carlo settings
☒	☐	For hierarchical and complex designs, identification of the appropriate level for tests and full reporting of outcomes
☒	☐	Estimates of effect sizes (e.g. Cohen's d, Pearson's r), indicating how they were calculated

Our web collection on statistics for biologists contains articles on many of the points above.

Software and code

Policy information about availability of computer code

Data collection	Excel
Data analysis	Excel, Statistica v. 13.5.0.17, MatLab

For manuscripts utilizing custom algorithms or software that are central to the research but not yet described in published literature, software must be made available to editors and reviewers. We strongly encourage code deposition in a community repository (e.g. GitHub). See the Nature Research guidelines for submitting code & software for further information.

Data

Policy information about availability of data

All manuscripts must include a data availability statement. This statement should provide the following information, where applicable:

- Accession codes, unique identifiers, or web links for publicly available datasets
- A list of figures that have associated raw data
- A description of any restrictions on data availability

Authors confirm that all relevant data are included in the paper and its supplementary information files.

Field-specific reporting

Life sciences study design

All studies must disclose on these points even when the disclosure is negative.

Sample size	The state-of-the-art standart size and sample numbers were taken for a particular technique (e.g., ≥ 4 for qRT-PCRs, ≥ 8 for metabolite measurements).
Data exclusions	No data were excluded from the analyses.
Replication	Phenotypic parameters of RNAi VPE2-repressed grains (1000 grain weight, grain width and length, grains per spike and starch content in the mature grains) were verified by analysing independent batches of plants growing at different time periods. Besides four lines, detailly analysed in the manuscript, additional homozygous lines were grown and phenotypic parameters of their grains were evaluated. RNAi-mediated repression of VPE2a, VPE2b and VPE2d genes was verified by qRT-PCR analysis of the grains from the independent batch of plants. All replicates were successful.
Randomization	All batches of growing plants were allocated by randomized block designs. The plants of each transgene line and the corresponding wild type were randomly organized within each of 4 or 8 sub-batches.
Blinding	Sample collection and sample analyses were performed by different researchers, specific details are described in the Materials and Methods section.

Reporting for specific materials, systems and methods

We require information from authors about some types of materials, experimental systems and methods used in many studies. Here, indicate whether each material, system or method listed is relevant to your study. If you are not sure if a list item applies to your research, read the appropriate section before selecting a response.

Materials & experimental systems

n/a	Involved in the study	
☒	☐	Antibodies
☒	☐	Eukaryotic cell lines
☒	☐	Palaeontology and archaeology
☒	☐	Animals and other organisms
☒	☐	Human research participants
☒	☐	Clinical data
☒	☐	Dual use research of concern

Methods

n/a	Involved in the study	
☒	☐	ChIP-seq
☐	☒	Flow cytometry
☒	☐	MRI-based neuroimaging

Flow Cytometry

Plots

Confirm that:

☐ The axis labels state the marker and fluorochrome used (e.g. CD4-FITC).

☐ The axis scales are clearly visible. Include numbers along axes only for bottom left plot of group (a 'group' is an analysis of identical markers).

☐ All plots are contour plots with outliers or pseudocolor plots.

☐ A numerical value for number of cells or percentage (with statistics) is provided.

Methodology

Sample preparation	Nuclei were isolated by manual chopping of the tissue with a sharp razor blade using nuclei isolation buffer according to Galbraith et al. (1983) supplemented with 50 µg ml-1 propidium iodide and 50 µg ml-1 DNase-free RNase and absorption distributions were measured on a Sysmex CyFlow Space flow cytometer (Sysmex-Partec, Germany). To estimate the cell number the number of nuclei was counted using the 'Absolute Cell Counting, function of the Sysmex CyFlow flow cytometer.
Instrument	Sysmec CyFlow Space flow cytometer (Sysmex-Partec, Germany)
Software	FloMax Operating and Analysis Software for Flow Cytometry Particle Analysing Systems, Version 2.82
Cell population abundance	Based on the applied settings the abundance of nuclei within the crude suspensions was above 90%.
Gating strategy	Nuclei were separated from cellular debris by plotting the log-scale relative DNA content against log-scale SSC signals.

☐ Tick this box to confirm that a figure exemplifying the gating strategy is provided in the Supplementary Information.
